# A Comparative Study on Relieving Exercise-Induced Fatigue by Inhalation of Different Citrus Essential Oils

**DOI:** 10.3390/molecules27103239

**Published:** 2022-05-18

**Authors:** Lei Tian, Tan Hu, Shanshan Zhang, Hongyan Zhang, Chenxi Yang, Guiting Chen, Siyi Pan

**Affiliations:** 1 College of Food Science and Technology, Huazhong Agricultural University, Wuhan 430070, China; tianlei@webmail.hzau.edu.cn (L.T.); hutan@webmail.hzau.edu.cn (T.H.); shaunazss@163.com (S.Z.); 15534064629@163.com (H.Z.); yangchenxi19990817@163.com (C.Y.); cgt@webmail.hzau.edu.cn (G.C.); 2 Key Laboratory of Environment Correlative Dietology, Huazhong Agricultural University, Ministry of Education, Wuhan 430070, China; 3 Hubei Key Laboratory of Fruit & Vegetable Processing & Quality Control, Huazhong Agricultural University, Wuhan 430070, China

**Keywords:** citrus essential oils, exercise-induced fatigue, oxidative stress, muscle injury, energy metabolism

## Abstract

Citrus essential oils (CEOs) possess physiological functions due to diverse aroma components. However, evidence for the effects of CEOs on exercise performance and exercise-induced fatigue is limited. The CEOs with discrepancies in components may exert different effects on the amelioration of exercise-induced fatigue. In this study, sweet orange (*Citrus sinensis* L.) essential oil (SEO), lemon (*Citrus limon Osbeck*) essential oil (LEO), and bergamot (*Citrus bergamia Risso and Poit*) essential oil (BEO) were chosen to explore the effect on amelioration of exercise-induced fatigue. Our results demonstrated that SEO and LEO increased the swimming time by 276% and 46.5%, while BEO did not. Moreover, the three CEOs exerted varying effects on mitigating exercise-induced fatigue via inhibiting oxidative stress, protecting muscle injury, and promoting glucose-dependent energy supply. Accordingly, BEO showed the best efficiency. Moreover, the GC-MS and Pearson correlation analysis of BEO showed that the contents of the major components, such as (±)-limonene (32.9%), linalyl butyrate (17.8%), and linalool (7.7%), were significantly positively correlated with relieving exercise-induced fatigue.

## 1. Introduction

With the increasing pressure in this fast-paced life, people are suffering from mental or physical fatigue due to irregular lifestyles. Physical fatigue, a universal and complex physiological phenomenon, is characterized by a temporary decrease in the ability of the body, which is caused by strenuous physical work, excessive exercise, or continuous long-term work or study [1]. Moreover, constant fatigue may further result in disease. Particularly, exercise-induced fatigue usually occurs after strenuous exercise, which seriously influences human health and work efficiency. The mechanisms of exercise-induced fatigue mainly include energy depletion, metabolite accumulation, oxidative stress, inflammatory responses, and excess reactive oxygen species (ROS)-mediated muscle injury [1]. An insufficient energy supply for muscle contraction by the depletion of glucose and glycogen leads to the fatigue. Besides, the accumulation of metabolites, such as lactic acid, caused by constant exercise contributes to changes in the homeostasis of the internal environment and an impairment of muscle contractility and therefore triggers muscle fatigue [2]. Moreover, strenuous exercise can excessively produce reactive oxygen species (ROS), resulting in oxidative stress of the body. Subsequently, the accumulation of ROS can damage cell membranes and trigger muscle injury [3]. In addition, the increased levels of proinflammatory cytokines can also induce inflammation and aggravate fatigue. Overall, the series of physiological responses eventually manifest as the loss of tissues and organs in the body, experienced as weakness and asthenia, and can even cause disease. Therefore, it is urgent to develop an agent and/or substances for ameliorating fatigue.

To date, various natural agents and/or substances have been utilized in preventing exercise fatigue since they are natural, environmentally friendly, and have low adverse effects on the body when compared with other ingredients. In particular, it has been demonstrated that some phytogenic active ingredients, especially polysaccharides [4], polyphenols [5], and polypeptides [6], exhibit excellent fatigue-relieving activities. Although significant efforts have been made to develop natural active ingredients with fatigue-relieving functions, it would be still beneficial to develop novel efficient compounds to overcome the fatigue induced by exercise. Essential oils (EOs) are composed of complex mixtures of virous secondary metabolites, such as terpenes (monoterpenes and sesquiterpenes), terpenoids, esters, alcohols, acids, aldehydes, and ketones [7]. They have been reported to possess physiological functions, including antioxidant, anti-inflammatory, and neuroprotective functions [8,9,10]. Recently, it has been demonstrated that EOs showed the effect of relieving or ameliorating fatigue in aromatherapy. Lin et al. [11] reported that the usage of aromatherapy by eucalyptus oil could relieve the fatigue of swimming rats by improving the swimming ability and antioxidant capacity in rats and then reducing tissue oxidative damage and the inflammatory response. Moreover, Li’s group [12] revealed that inhalation of an essential oil mixture composed of *Citrus sinensis* L., *Mentha piperita* L., *Syzygium aromaticum* L. and *Rosmarinus officinalis* L. exhibited an excellent anti-fatigue effect on physical exhaustion in rats. The mixed oils can significantly increase the content of blood glucose and decrease that of blood urea nitrogen (BUN) and glutathione peroxidase (GSH-PX) in rats. Moreover, it has been reported that citrus essential oil is known for its calming and soothing properties that relax the muscles [13,14]. Besides, citrus essential oils (CEOs) are one of the most important categories of EOs and are mainly derived from the oil sac of citrus exocarp, flowers, leaves, and other tissues [15]. Due to its pleasant fragrance and recognized safety [16], citrus essential oil is widely applied in food, cosmetics, and medicine. Lemon essential oil was proven to be effective in reducing lipid peroxidation levels and increasing the superoxide dismutase (SOD), catalase (CAT), and glutathione peroxidase (GSH-Px) activities in the mouse hippocampus [13]. d-limonene was revealed to be able to protect oxidative-stress-induced cell damage in human lens epithelial cells via the p38 pathway [17]. Meanwhile, the high d-limonene content of CEOs, such as sweet orange and bergamot essential oil, was demonstrated to have a strong free radical scavenging ability and exhibits antioxidant activity [9,17,18,19]. Interestingly, Nagai et al. [20] found that lavender essential oils stimulated skeletal muscle sympathetic excitability through olfactory activity, which increased oxygen and nutrient supplies to the skeletal muscles and helped them recover from fatigue and promoted muscle growth. Notably, the major components of lavender oil are linalyl acetate, linalool, β-ocimene, and β-caryophyllene, which is very similar to the composition in bergamot essential oil [21]. We speculated that the CEOs could also relieve exercise fatigue. However, the effects of different varieties of CEOs and the underlying mechanisms on the fatigue induced by exhaustive exercise have not yet been investigated.

The contents and chemical compositions of CEOs are dependent on the citrus species, parts of citrus plants, climate of origin, season, growth stage, and extraction method [22]. To establish the correlations between key components of different CEOs and fatigue-relieving efficacy, in this work, sweet orange essential oil (SEO), lemon essential oil (LEO), and bergamot essential oil (BEO) were selected to investigate the effect of the inhalation of the CEOs (SEO, LEO, and BEO) on alleviating the fatigue induced by exhaustive swimming. We studied the effects of the three CEOs with different chemical components on relieving exercise-induce fatigue. Then, we focused on the energy supply, metabolism accumulation, oxidative stress, and muscle injury in exhaustive-swimming-induced fatigue rats in order to explore their underlying mechanisms. This work can provide a novel insight into aromatherapy for mitigating exercise-induced fatigue and suggestions for the exploitation of safe exercise foods for physical efficiency and exercise performance.

## 2. Results

### 2.1. Chemical Composition of the CEOs

The chemical composition of the CEOs, observed by SPME/GC-MS analysis, is shown in Table 1. A total of 26 components were found in SEO. The major components were (±)-limonene (81.7%), β-myrcene (4.6%), linalool (2.8%), decanal (2.5%), and β-terpinene (2.2%). Nearly 30 components were detected in LEO, and the major components were (±)-limonene (38.6%), citral (12.1%), β-terpinene (11.6%), γ-terpinene (7.7%), and neryl acetate (4.2%). A total of 24 components were detected in BEO. The major components were (±)-limonene (33,0%), linalyl butyrate (17.8%), β-terpinene (10.0%), γ-terpinene (8.2%), and linalool (7.7%). It could be observed that the relative content of (±)-limonene in the SEO (81.7%) was significantly greater than that in LEO (38.6%) and BEO (32.9%). Moreover, we also found more oxygenated compounds in LEO (29.2%) and BEO (29.5%) than in SEO (7.0%). Interestingly, it was also noted that the relative content of linalyl butyrate was 17.8% in BEO, which is inconsistent with the value reported in other studies [21]. It was generally reported that linalyl acetate was the second most abundant compound in BEO [15]. This could be partly attributed to the origin and harvest time of the fruits and the analytical techniques [21].

### 2.2. Effect of the Inhalation of CEOs on Physiological Indices in Rats

**Figure 1:**
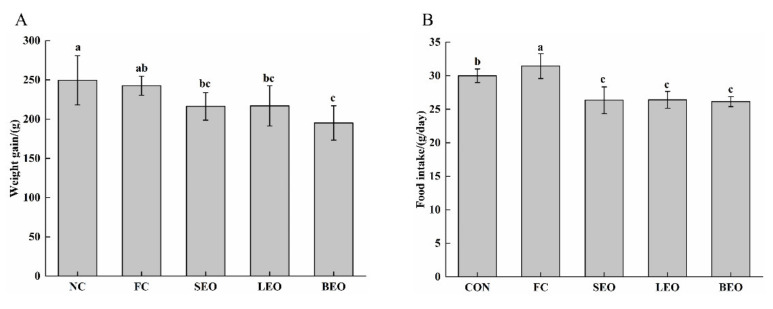
Effects of inhalation of the CEOs on weight gain and average food intake in rats. (**A**) weight gain, (**B**) average food intake. Data are the means ± SD. Different lowercase letters indicate significant differences, *p* < 0.05.

The weight gain and average food intake of rats are presented in Figure 1.

Compared with the FC group, the weight in the BEO group was significantly reduced (*p* < 0.05) from 242 ± 12 to 195 ± 22 g. Besides, the food intake of the SEO, LEO, and BEO groups significantly decreased (*p* < 0.05) by 20.4%, 22.2%, and 20.7%, respectively. These results suggest that the inhalation of the CEOs could inhibit appetite and control the body weight of rats, especially BEO.

The organ indices of rats are shown in Table 2. There were no significant differences among the FC and CEOs groups in the liver and spleen indices (*p* > 0.05). However, the kidney indices significantly increased in the CEO groups (*p* < 0.05) compared with the FC group. The results demonstrated that the inhalation of the CEOs resulted in an increase in kidney weight.

### 2.3. Effect of the Inhalation of CEOs on Exercise Performance in Rats

**Figure 2:**
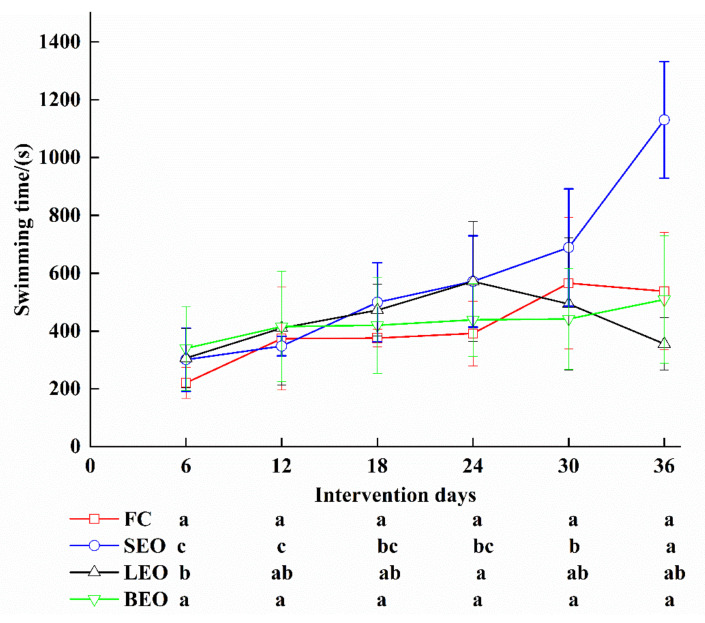
Effects of inhalation of the CEOs on swimming exhaustion time in rats. Data are the means ± SD. Different lowercase letters indicate significant differences, *p* < 0.05.

In order to explore the effect of the inhalation of the CEOs on exercise performance, weight-loaded swimming trials were conducted with the FC and CEO groups every six days. The swimming duration of each trial is shown in Figure 2. Interestingly, the swimming duration in the SEO group increased significantly with the time of inhalation treatment, where the swimming duration increased by 276.15% on the 36th day compared to that on the 6th day (*p* < 0.05). The swimming duration in the LEO group increased and then decreased during the period of trails, where the longest duration was 571 ± 208 s on the 24th day and significantly increased by 46.45% when compared to that on the 6th day (*p* < 0.05). However, there were no significant increases in the FC and BEO groups among the six trials (*p* > 0.05). On the other hand, on the 36th day, the swimming duration of the SEO group significantly increased when compared with the FC group (*p* < 0.05). These results indicate that both SEO and LEO can prolong the swimming duration in rats.

### 2.4. Effect of the Inhalation of CEOs on Energy Supply in Rats

**Figure 3:**
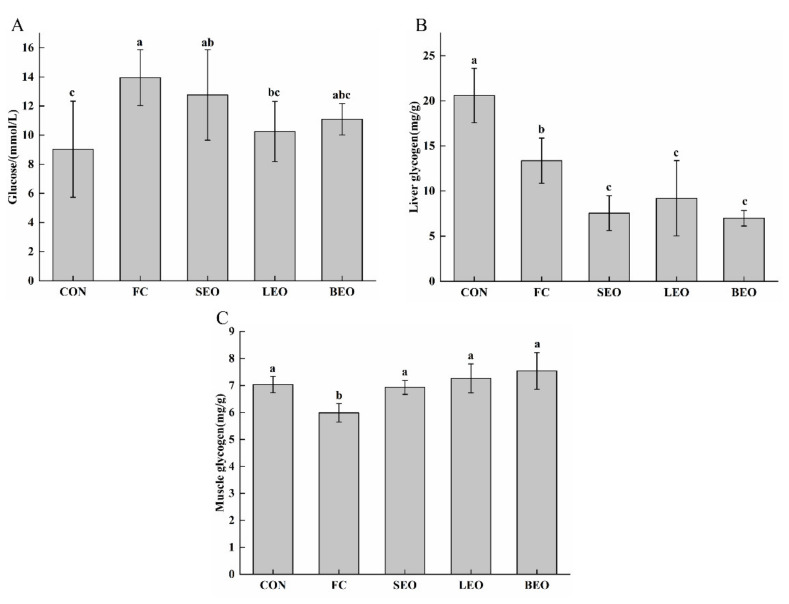
Effects of the inhalation of the CEOs on energy supply in exercise-induced fatigue rats. (**A**) Glucose in serum, (**B**) LG in liver, (**C**) MG in gastrocnemius. Data are the means ± SD. Different lowercase letters indicate significant differences, *p* < 0.05.

Blood samples were collected immediately after exhaustive swimming. Glucose is the direct supplier of energy [23]. As shown in Figure 3A, the content of blood glucose in the FC group (13.95 ± 1.92 mmol/L) increased significantly compared with the CON group (9.03 ± 3.30 mmol/L) (*p* < 0.05). Compared with the FC group, the glucose content in the LEO group significantly decreased by 26.52% (*p* < 0.05) but showed no significant difference from the CON group.

Glycogen, a stored form of glucose, plays an important role in energy supply during exercise [24]. Energy supply is essential for muscle contraction [23]. The liver and muscle glycogen levels are depicted in Figure 3B,C. The levels of LG in the CEO groups significantly decreased (by 43.53%, 31.19%, and 47.72%, respectively) when compared with the FC group (*p* < 0.05). The levels of LG and MG significantly decreased after exhaustive swimming in the FC group compared to the CON group (*p* < 0.05). The MG in the CEO groups increased significantly compared to the FC group (*p* < 0.05) and showed no significant difference from the CON group (*p* > 0.05). These results suggest that the CEOs could promote glycogenolysis, regulate blood glucose during exercise, and enhance muscle glycogen storage for the body to mitigate fatigue.

### 2.5. Effect of the Inhalation of CEOs on Metabolite Accumulation in Rats

**Figure 4:**
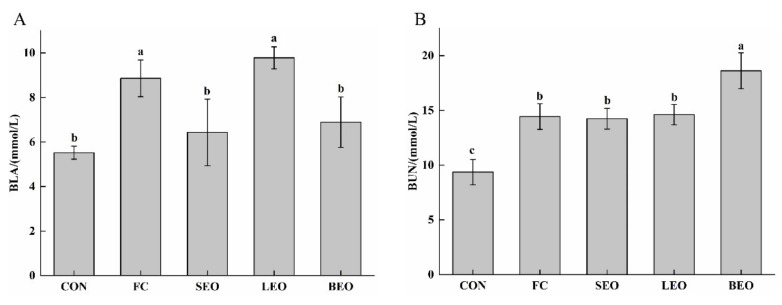
Effects of the inhalation of the CEOs on metabolite accumulation in exercise-induced fatigue rats. (**A**) BLA in serum, (**B**) BUN in serum. Data are the means ± SD. Different lowercase letters indicate significant differences, *p* < 0.05.

BLA and BUN are important indicators for the severity of fatigue [1]. As shown in Figure 4A, the content of BLA in the SEO and BEO groups significantly decreased from 8.86 ± 0.82 to 6.43 ± 1.49 and 6.89 ± 1.13 mmol/L (*p* < 0.05) when compared to that in FC group. Meanwhile, the BLA contents in SEO and BEO groups showed no significant differences compared to the CON group. However, there was no significant difference for the BUN content (Figure 4B) among the SEO, LEO, and FC groups (*p* > 0.05). On the contrary, the BUN content of the BEO group increased significantly when compared with that of the FC group (*p* < 0.05). The results indicate that daily inhaling of SEO and BEO could inhibit the accumulation of lactic acid and/or scavenge lactic acid.

### 2.6. Effect of the Inhalation of CEOs on Oxidative Stress in Rats

**Figure 5:**
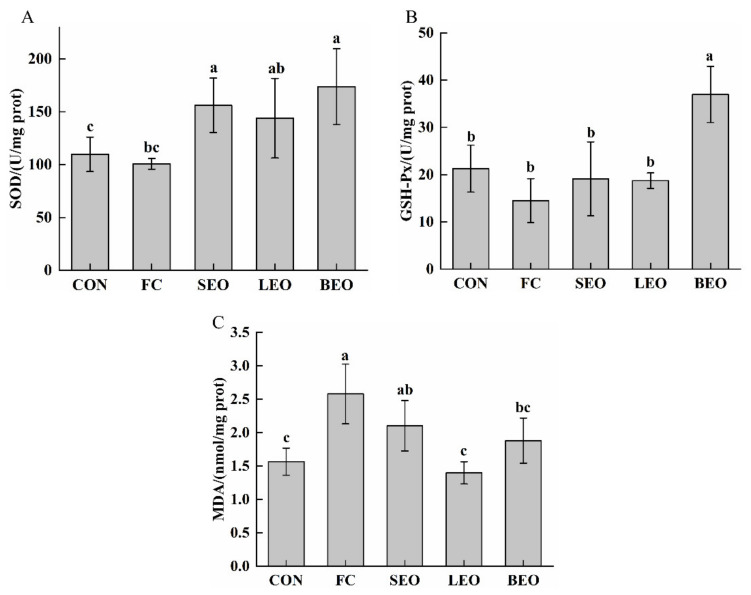
Effects of inhalation of the CEOs on oxidative stress in exercise-induced fatigue rats. (**A**) SOD in gastrocnemius, (**B**) GSH-Px in gastrocnemius, (**C**) MDA in gastrocnemius. Data are the means ± SD. Different lowercase letters indicate significant differences, *p* < 0.05.

Strenuous-exercise-induced oxidative stress can excessively produce reactive oxygen species (ROS) during cellular respiration, which can trigger peripheral fatigue [1]. Superoxide dismutase (SOD) and glutathione peroxidase (GSH-PX) are important endogenous antioxidant enzymes that are included in the intracellular defense systems to scavenge free radicals [25]. As shown in Figure 5A, the levels of SOD in the SEO (156.09 ± 25.73 U/mg prot) and BEO (173.78 ± 35.98 U/mg prot) groups were significantly higher than that in the FC group (100.74 ± 5.04 U/mg prot) (*p* < 0.05). Interestingly, only the level of GSH-Px in the BEO group significantly increased compared to that in the FC group (*p* < 0.05, Figure 5B), from 14.51 ± 36.95 to 36.95 ± 5.95 U/mg prot. The GSH-Px levels in the SEO and LEO groups showed no significant differences compared to the FC group (*p* > 0.05). Malondialdehyde (MDA) can reflect potential antioxidant capacity, which is frequently utilized to evaluate body fatigue severity in combination with antioxidant enzymes, such as SOD and GSH-Px. As shown in Figure 5C, the inhalation of LEO and BEO significantly reduced the exhaustive-swimming-induced rise in MDA (*p* < 0.05) by 45.74% and 27.13%. Therefore, these results indicate that SEO could upregulate SOD activity, and LEO can reduce MDA content. BEO could effectively upregulate SOD and GSH-Px levels and attenuate lipid peroxidation intensity, suggesting that BEO could alleviate fatigue via the inhibition of oxidative stress.

### 2.7. Effect of the Inhalation of CEOs on Muscle Injury in Rats

**Figure 6:**
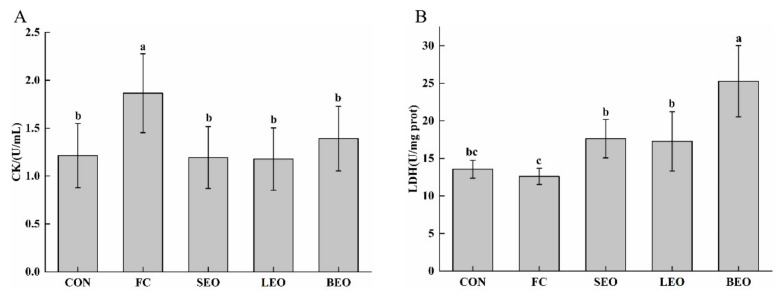
Effects of the inhalation of the CEOs on muscle injury in exercise-induced fatigue rats. (**A**) CK in serum, (**B**) LDH in gastrocnemius. Data are the means ± SD. Different lowercase letters indicate significant differences, *p* < 0.05.

Insufficient supplementation of energy and an accumulation of ROS during strenuous exercise leads to an increased permeability and even disintegration of the muscle cell membrane, causing creatine kinase (CK) in the myocytes to flow into the blood [1]. As shown in Figure 6A, the three CEOs significantly decreased the level of CK compared with the FC group (*p* < 0.05), by 36.36%, 36.90%, and 25.67% from 1.87 ± 0.41 to 1.19 ± 0.32, 1.18 ± 0.33, and 1.39 ± 0.34 U/mg prot, respectively.

Lactate dehydrogenase (LDH) is an enzyme that catalyzes the reciprocal conversion of pyruvate and lactic acid [25]. It was reported that the LDH concentration induced by intense exercise may cause muscle damage, and LDH was regarded as a specific marker of the disruption of muscle fibers [26]. As shown in Figure 6B, compared with the FC group, the activity of LDH in the gastrocnemius significantly increased by 39.9%, 37.0%, and 100.1% from 12.60 ± 1.08 to 17.63 ± 2.56, 17.27 ± 3.96, and 25.27 ± 4.75 U/mg prot (*p* < 0.05), respectively. These findings demonstrated that the CEOs can alleviate muscle injuries induced by exhaustive exercise, with BEO being the best.

**Figure 7:**
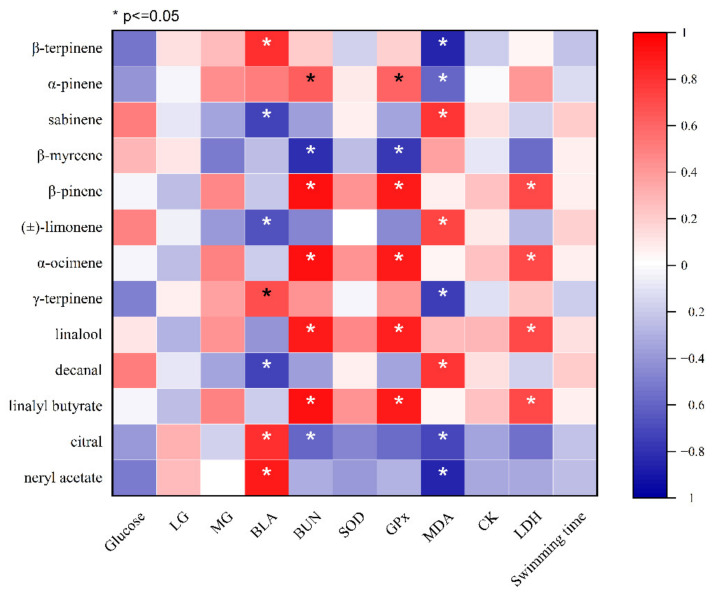
Pearson correlation analysis between exercise performance, fatigue-associated oxidative stress, energy supply, metabolite accumulation, and muscle injury indices. Blue color indicates a negative correlation, and red color indicates a positive correlation. * indicates a significant correlation between groups (*p* < 0.05).

A Pearson correlation was used to analyze the relationship between exercise performance, fatigue-associated energy supply, metabolite accumulation, oxidative stress, muscle injury indices, and the major components of the CEOs (relative contents more than 1%). As shown in Figure 7, the BLA level was negatively correlated with decanal, sabinene, and (±)-limonene and was positively correlated with the relative contents of β-terpinene, γ-terpinene, citral, and neryl acetate (*p* < 0.05). BUN was negatively correlated with β-myrcene and citral, while it was positively correlated with β-pinene, α-ocimene, linalool, linalyl butyrate, and α-pinene (*p* < 0.05). GSH-Px was positively correlated with β-pinene, α-ocimene, linalool, linalyl butyrate, and α-pinene, while it was negatively correlated with β-myrcene (*p* < 0.05). MDA was negatively correlated with neryl acetate, β-terpinene, γ-terpinene, citral, and α-pinene, while it was positively correlated with sabinene, decanal, and (±)-limonene (*p* < 0.05). LDH was positively correlated with linalool, β-pinene, linalyl butyrate, and α-ocimene (*p* < 0.05). Based on the results, we suggest that SEO could strongly improve the reduction of metabolites during exercise. BEO could strongly alleviate fatigue by inhibiting oxidative stress and protecting from muscle injury. LEO could weakly relieve fatigue by reducing metabolites and inhibiting oxidative stress.

## 3. Discussion

CEOs have been demonstrated to possess antioxidant, anti-inflammatory, neuroprotective, antibacterial, and other physiological activities due to diverse and complex active components. It has been reported that the chemical composition and contents of CEOs are related to citrus varieties and the method of extraction and separation, which might determine the differences in functional properties [27]. From the GC-MS results (Table 1), the highest limonene content was found in SEO compared to that in LEO and BEO. However, according to the results of the fatigue-relieving-related indicators, BEO showed the best efficiency, suggesting that limonene was not the key factor for alleviating fatigue. In order to further understand the effect of different species of CEOs on relieving fatigue and its underlying mechanism, we also analyzed the minor components of CEOs. The results showed more oxygenated compounds in LEO and BEO than that in SEO. Moreover, BEO contained more linalyl butyrate and linalool than LEO. The Pearson correlation results (Figure 7) suggested that the linalyl butyrate and linalool in BEO were vital for alleviating fatigue.

The mechanism of fatigue is extremely complex. There are many factors causing fatigue, such as insufficient energy supply, the accumulation of metabolites, oxidative stress, muscle injury, and inflammation [1]. We established an ideal fatigue experimental model by a weight-loaded exhaustive swimming test to reflect the exercise performance in rats. The energy supply, metabolite accumulation, oxidative stress, and muscle injury were used to evaluate the effects of the CEOs on alleviating the fatigue induced by exhaustive swimming. The results showed that the daily inhalation of SEO and LEO could significantly improve the swimming duration of rats. We also found that the food intake and weights of the CEO treatment rats were inhibited significantly, indicating the inhibition of the CEOs in the appetite and weight gain of rats. This could be explained by the fact that CEOs can activate the sympathetic nerve, which innervates white adipose tissue (WAT), which promotes lipolysis and thereby inhibits weight gain [28]. Hence, in order to further explore the fatigue-relieving ability of the CEOs, biochemical indices related to fatigue in the serum and gastrocnemius were also determined.

Effective ATP resynthesis is needed to match the dramatically increased ATP consumption in response to strenuous exercise [29]. Liver glycogen, muscle glycogen, and blood glucose are related to energy supply during exercise [5,30]. High-intensity strenuous exercise can activate the sympathetic nervous system of the body. Subsequently, adrenaline, glucagon, glucocorticoids, and other hormones are secreted, which promote liver glycogenolysis, resulting in an increase in blood glucose [31]. From the results of blood glucose, it can be noted that the content of glucose in the FC group was higher than that in the CON group, which is inconsistent with other studies. It was reported that blood glucose increases during intense exercise and increases further immediately at exhaustion and persists for up to 1 h [32]. Since we sacrificed rats immediately after swimming procedure. The decomposition of liver glycogen produced glucose after exhaustive exercise. Our hypothesis was that the rate of glucose output exceeded the consumption rate during exercise, leading to an increase in blood glucose. In this study, we found that there was a decrease in LG (Figure 3B) and glucose (Figure 3A) and an increase in MG (Figure 3C) that were treated by the inhalation of the CEOs when compared with those in the FC group. It might be because the CEOs promoted glycogenolysis, MG storage, and/or gluconeogenesis restraint. This can be partly attributed to insulin secretagogue bioactivity of the bioactive compounds in CEOs, which were D-limonene in SEO, LEO, and BEO (81.65%, 38.63%, and 32.92%, respectively) and β-pinene in BEO (4.62%). These components were demonstrated to be key factors in using citrus fruit to promote insulin secretion and show effective hypoglycemic activity [33]. Besides, the simultaneous increases in glucose and insulin concentrations create a milieu that should favor the repletion of at least part of the muscle glycogen that is mobilized [34]. Therefore, it implied that the CEOs could promote glycogenolysis and the uptake of glucose by skeletal muscle through the glycolysis pathway to quickly supply energy during exercise, contributing to the preservation of muscle glycogen. These results suggest that CEOs could promote glucose-dependent energy supply during exhaustive exercise and enhance muscle glycogen storage to alleviate fatigue.

The accumulation of metabolites is detrimental to muscle contraction [23] and induces the development of fatigue. Strenuous exercise leads to a hypoxic state in the body. Carbohydrates are converted into lactic acid via anaerobic oxidation in the glycolysis pathway and then cause an increase in the content of BLA [1]. With the increase in lactic acid, the increasingly dissociated H^+^ will disturb the body homeostasis and thus affect the metabolism of the body. On the other hand, proteins and amino acids are decomposed during exercise, causing the accumulation of BUN [12]. In addition, insufficient O_2_ in mitochondria leads to an inadequate oxidation reaction and lower energy generation as well as the accumulated metabolites [23]. Kang et al. [35] reported that BEO could induce vasorelaxation in mice and thus enhance oxygen and nutrient supplies for the skeletal muscle. Our findings showed that the inhalation of SEO and BEO effectively reduced the content of BLA. Excessive BLA in the SEO and BEO groups were converted to pyruvate that was catalyzed by LDH. Consequently, pyruvate continued to be oxidized in mitochondria and then underwent the citric acid cycle and oxidative phosphorylation to release more energy. However, the CEO administration failed to decrease the content of BUN. Of note, BUN is also an indicator to reflect the state of the kidneys, which are important organs to remove metabolites. This may explain the increased kidney indices in the CEO groups. Overall, we suggest that the inhalation of SEO and BEO can improve the mitochondrial function to supply energy for the body during exercise. The CEOs could not alleviate fatigue by the adjustment of the BUN level.

Fatigue is closely correlated with oxidative stress. Strenuous exercise leads to an oxidant–antioxidant imbalance and generates superfluous free radicals, such as ROS, further contributing to muscle fatigue. SOD and GSH-Px play crucial roles in protecting cells from oxidative stress by scavenging free radicals and their metabolites, regulating the equilibrium between oxidation and antioxidation. Besides, MDA can reflect the potential antioxidant capacity, the rate and intensity of lipid peroxidation, and the severity of tissue peroxidation damage [4]. It can be noted that BEO could increase the level of SOD and GSH-Px and decrease the MDA content. A previous study demonstrated that a *C. lumia* EO that was rich in d-limonene and linalool acted as a chelating agent to reduce the availability of transition metals, to lower Fenton-like oxidative chain reactions in biological systems, and to preserve the integrity and functionality of membranes [36]. Moreover, the antioxidant compounds in the EO can form adducts with peroxyradicals to inhibit lipid peroxidation with strong antioxidant activity. Additionally, it has been reported that α-pinene and β-pinene possess an antioxidant activity via reducing nitric oxide production [37]. Since oxidative stress can trigger an inflammatory response. Linalool and linalyl acetate were further demonstrated as potential anti-inflammatory activities in another study [38]. Our results showed the contents of α-pinene (5.1%) and β-pinene (4.62%) in BEO were higher than those in SEO (0.03% and 0.09%, respectively) and LEO (3.57% and ND, respectively). Besides, BEO contains 17.83% linalyl butyrate, which shows similar chemical properties because of the homolog of linalyl acetate [39]. Therefore, the results indicated that BEO can better inhibit the oxidative stress induced by exhaustive exercise than SEO and LEO. This can be partly explained by the synergistic effect of (±)-limonene, linalool butyrate, linalool, α-pinene, β-pinene, and other minor components with strong antioxidant activities in BEO that may enhance its fatigue-relieving activity.

The accumulation of ROS produced by oxidative stress may affect cell membranes. Slight muscle injury by excessive exercise will induce decreased muscle strength [1]. Both LDH and CK are important indicators of muscle injury. The increased permeability or even disintegration of myocyte membranes cause CK and LDH to flow into the bloodstream [1]. The data showed that the three CEOs could effectively reduce the level of CK. We noticed that the activity of LDH in the CEOs increased in the gastrocnemius where the level in BEO was the greatest. This may attributed by the high content of linalyl butyrate and linalool, which is in agreement with Nagai’s results [20]. Our finding demonstrated that the CEOs could alleviate fatigue via protection from muscle injury.

Based on the aforementioned results, BEO showed efficient relieving of exhaustive-exercise-induced fatigue activity. However, no significant increase was observed in swimming exhaustion time treated by BEO, which might be due to its sedative activity [40]. Although SEO and LEO could prolong the duration of swimming in rats at different intervention days, they showed inefficient mitigation of exhaustive-exercise-induced fatigue activity when compared with BEO. This might due to the excellent analgesic activity of BEO and LEO [41,42]. The specific underlying mechanism of the phenomena remains to be further explored.

## 4. Materials and Methods

### 4.1. Materials

SEO, LEO, and BEO (the CEOs, Capua, Italy) were bought from Beijing Zoteq Co., Ltd. (Beijing, China). The assay kits, including blood lactic acid (BLA), blood urea nitrogen (BUN), creatine kinase (CK), glucose, liver glycogen (LG), muscle glycogen (MG), lactate dehydrogenase (LDH), superoxide dismutase (SOD), glutathione peroxidase (GSH-Px), and malondialdehyde (MDA), were bought from Nanjing Jiancheng Bioengineering Institute (Nanjing, China). All other chemical reagents for this investigation were of analytical grade and were bought from Sinopharm Chemical Reagent Co., Ltd (Shanghai, China).

### 4.2. GC-MS

The analysis method was slightly modified based on a previous study [43]. The volatile compounds of the CEOs were identified and quantified by solid-phase microextraction (SPME)/GC-MS. SPME was conducted by a manual device equipped with a 50/30 μm divinylbenzene (DVB)/carboxen (CAR)/polydimethylsiloxane (PDMS) fiber (Supelco, Bellefonte, PA, USA). The samples were maintained in an enclosed headspace bottle with a volume of 25 mL, which was covered with 0.5 mL of the three CEOs and 3 mL of anhydrous ethanol. The fiber was exposed for 40 min to extract the volatile constituents after 15 min of water-bath heating equilibration at 40 °C. Then, the fiber was inserted through a septum and the injection port of GC for 5 min.

The solid-phase microextraction (SPME) extracts were analyzed using an Agilent 7890B GC coupled with an Agilent 5977A mass spectrometer (Agilent Technologies, Palo Alto, CA, USA). Analytes were separated using an HP-5 fused silica capillary column (30 × 320 × 0.25 μm). The conditions of GC-MS were slightly modified from the method of Tai [43]. The experiments were performed in triplicate.

The components of the samples were compared with the National Institute of Standards and Technology (NIST) 05 (Gaithersburg, MD, USA) and Wiley 7.0 (Hoboken, NJ, USA) libraries with a similarity of more than 80%. The concentrations are presented as percentages of the area under each peak relative to the total area.

### 4.3. Experimental Animals

Forty special pathogen-free male Sprague Dawley rats (weight 160~180 g, Hunan SJA Laboratory animals Co., Ltd., Hunan, China) were maintained in the Experimental Animal Centre of Huazhong Agricultural University with 12 h light/dark cycles at a stationary temperature of 24 ± 2 °C and a relative humidity of 40–70%. All rats were housed as four rats per cage with standard food and tap water ad libitum.

### 4.4. Animal Experiment Design

**Figure 8:**
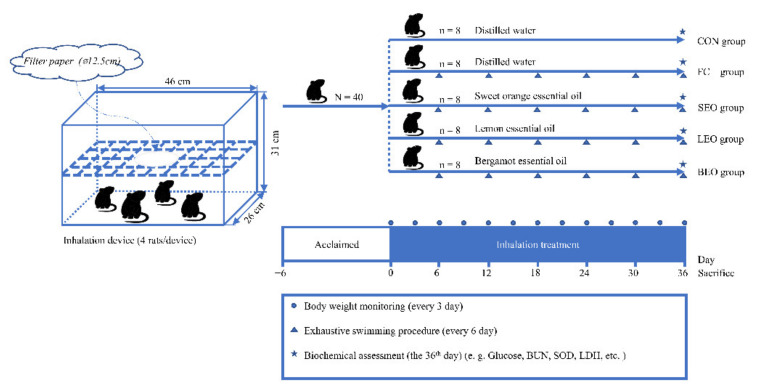
Experimental procedure for the effects of inhalation of the CEOs on exercise-induced fatigue.

The animals were acclimated to the new environment for 6 days (Figure 8). During the last three days of the adaptive feeding period, swim training was performed for 20 min each day. All rats were able to swim. After that, the animals were randomly divided into five groups (*n* = 8 per group): control group (CON), fatigue control group (FC), sweet orange essential oil group (SEO), lemon essential oil group (LEO), and bergamot essential oil group (BEO). The CEO groups were administrated SEO, LEO, and BEO for 2 h once daily at a dose of 1 mL per cage for 36 days. The CON and FC groups were treated with distilled water only. The inhalation of the CEOs or distilled water was conducted in manual devices, which were semi-enclosed spaces (46 × 31 × 26 cm) with partitions. The filter papers (⌀ 12.5 cm) with the CEOs or distilled water were placed on the central partition to volatilize and distribute evenly at room temperature (24 ± 2 °C). The measured volatile rate of the CEOs was 71.97 ± 0.01%. The weight gain and food intake were recorded. 

### 4.5. Exhaustive Swimming Test

The CEO and FC groups were subjected to exhaustive swimming every six days. After the last inhalation procedure, the CEO and FC groups were moved into a swimming pool for forced swimming to exhaustion. The control group received no treatment at the end of the experiment. The pool was made of a plastic chamber of 81 cm length, 60 cm width, and 59 cm height filled with water up to 50 cm and maintained at 28 ± 1 °C. In addition, the weights on the tail root of each rat corresponded to 7% of body weight as evaluated. The swimming time was recorded immediately when the rats were submerged in water for more than 7 s three times and failed to rise to the water surface for breathing, which was determined as the exhaustion criteria. The water in the swimming pool was refreshed and the temperature was readjusted after each session. At the end of every exhaustive swimming test, the rats were dried with paper towel and were taken back to their cages to rest.

### 4.6. Determination of Fatigue-Associated Biochemical Parameters

After exhaustive swimming, the rats were anesthetized with ether, and blood samples were collected immediately from the eyeball. Serum was obtained by centrifugation at 1123× *g* and 4 °C for 10 min and was kept at −20 °C for further study. Rats were sacrificed after blood collection. Livers, spleens and kidneys were weighed to calculate organ indices (organ indices (%) = organ/body weight (g) × 100). The gastrocnemius and liver were kept at −80 °C for further analysis. The serum indicators (BLA, BUN, glucose, and CK), liver indicator (LG), and muscle indicators (MG, LDH, SOD, GSH-PX, and MDA) were determined using the kits according to the respective protocols.

### 4.7. Statistical Analysis

All the experimental results were expressed as means ± SD. The statistical significance (*p* < 0.05) was calculated using a one-way ANOVA followed by Duncan’s test using IBM SPSS Statistics 26 (IBM Corp., Armonk, NY, USA). There were significant differences among the groups, represented by different letters in the figures (*p* < 0.05). Origin 2021(OriginLab Corp., Northampton, MA, USA) and Microsoft PowerPoint 2019 (Microsoft Corp., Redmond, WA, USA) were used for mapping.

## 5. Conclusions

In this work, we focus on the effect of the CEOs on alleviating exercise-induced fatigue. The inhalation of SEO and LEO can significantly prolong the swimming time. Importantly, BEO, LEO, and SEO all mitigate exhaustive-exercise-induced fatigue via the inhibition of oxidative stress, the protection of muscle injury, and the promotion of glucose-dependent energy supply. BEO showed the greatest ability to relieve fatigue, while SEO was inefficient. This can be explained by the different major components in the CEOs that showed discrepancies in physiological activities. Our findings can provide a novel insight into aromatherapy to ameliorate exercise fatigue using natural essential oils and suggestions for the exploitation of safe exercise foods for physical efficiency and exercise performance.

## Figures and Tables

**Table 1:** Chemical composition of sweet orange (*Citrus sinensis* L.), lemon (*Citrus limon Osbeck*), and bergamot (*Citrus bergamia Risso and Poit*) essential oils.

No.	RT/(min)	Compound	Molecular Formula	Relative Content (%)		
				SEO	LEO	BEO
1	10.52	β-terpinene	C_10_H_16_	2.18	11.56	9.95
2	11.32	tricyclene	C_10_H_16_	ND	ND	0.15
3	11.93	α-pinene	C_10_H_16_	0.03	3.57	5.10
4	12.06	β-phellandrene	C_10_H_16_	0.02	ND	ND
5	12.60	sabinene	C_10_H_16_	1.76	ND	ND
6	12.62	β-thujene	C_10_H_16_	ND	0.11	ND
7	13.33	*p*-cymene	C_10_H_14_	ND	ND	0.01
8	13.72	β-myrcene	C_10_H_16_	4.62	2.51	ND
9	13.77	β-pinene	C_10_H_16_	0.09	ND	4.62
10	14.05	sylvestrene	C_10_H_16_	ND	ND	0.61
11	15.85	(±)-limonene	C_10_H_16_	81.65	38.63	32.92
12	16.69	α-ocimene	C_10_H_16_	ND	ND	2.94
13	16.73	(+)-3-carene	C_10_H_16_	0.21	0.35	ND
14	17.17	γ-terpinene	C_10_H_16_	1.20	7.68	8.15
15	17.51	*trans*-isolimonene	C_10_H_16_	ND	ND	0.08
16	18.42	α-terpinolene	C_10_H_16_	ND	0.45	0.91
17	18.44	2-carene	C_10_H_16_	0.11	ND	ND
18	19.03	sabinene hydrate	C_10_H_18_O	ND	0.04	ND
19	19.43	nonanal	C_9_H_18_O	0.14	ND	ND
20	19.47	linalool	C_10_H_18_O	2.77	0.89	7.67
21	19.48	1-cyclopropylpentane	C_8_H_16_	ND	0.08	ND
22	21.10	(+)-2-bornanone	C_10_H_16_O	ND	ND	0.05
23	21.19	1,3,8-*p*-menthatriene	C_10_H_14_	ND	0.08	ND
24	21.30	β-fenchol	C_10_H_18_O	ND	0.04	ND
25	21.70	*trans*-*p*-mentha-2,8-dienol	C_10_H_16_O	ND	0.08	ND
26	22.20	neo-allo-ocimene	C_10_H_16_	ND	0.01	0.28
27	22.28	limonene 1,2-epoxide	C_10_H_16_O	0.03	0.05	0.02
28	23.42	citronellal	C_10_H_18_O	0.09	0.19	ND
29	22.44	1*R*,4*R*-*p*-mentha-2,8-dien-1-ol	C_10_H_16_O	ND	0.20	ND
30	23.66	pinocarvone	C_10_H_14_O	ND	0.04	ND
31	24.46	(−)-terpinen-4-ol	C_10_H_18_O	ND	0.12	ND
32	24.43	decanal	C_10_H_20_O	2.53	ND	ND
33	24.71	cyclooctane	C_8_H_16_	0.02	ND	ND
34	25.20	α-terpineol	C_10_H_18_O	ND	0.35	ND
35	26.04	*cis*-citral	C_10_H_16_O	0.48	6.41	0.13
36	26.47	linalyl butyrate	C_14_H_24_O_2_	ND	ND	17.83
37	27.39	*trans*-citral	C_10_H_16_O	0.87	ND	0.06
38	27.48	*cis*-carveol	C_10_H_16_O	ND	0.20	ND
39	28.25	3-cyclohexen-1-one, 2-isopropyl-5-methyl-	C_10_H_16_O	ND	0.06	ND
40	29.60	citral	C_10_H_16_O	ND	12.09	ND
41	30.84	α-terpinyl acetate	C_12_H_20_O_2_	ND	ND	0.05
42	31.67	neryl acetate	C_12_H_20_O_2_	0.07	4.17	1.48
43	32.55	geranyl acetate	C_12_H_20_O_2_	ND	4.05	2.24
44	32.48	bicyclo [4.4.0] dec-1-ene, 2-isopropyl-5-methyl-9-methylene-	C_15_H_24_	0.10	ND	ND
45	32.59	beta-elemene	C_15_H_24_	0.04	ND	ND
46	33.47	dodecanal	C_12_H_24_O	0.02	ND	ND
47	33.70	caryophyllene	C_15_H_24_	0.13	1.32	0.34
48	34.09	α-cubebene	C_15_H_24_	0.14	ND	ND
49	34.46	α-bergamotene	C_15_H_24_	ND	0.07	0.11
50	36.77	(+)-valencene	C_15_H_24_	0.38	ND	ND
51	38.04	δ-cadinene	C_15_H_24_	0.06	ND	ND
52	39.18	β-bisabolene	C_15_H_24_	ND	0.09	0.04

RT, Retention time; ND, not detected.

**Table 2:** Effects of inhaling the CEOs on organ indices in rats.

Groups	Organ Indices (%)		
	Liver Indices	Spleen Indices	Kidney Indices
CON	3.85 ± 0.34 ^a^	0.16 ± 0.02 ^a^	0.67 ± 0.06 ^b^
FC	3.56 ± 0.20 ^ab^	0.17 ± 0.02 ^a^	0.65 ± 0.03 ^b^
SEO	3.40 ± 0.28 ^b^	0.17 ± 0.03 ^a^	0.74 ± 0.03 ^a^
LEO	3.43 ± 0.40 ^b^	0.15 ± 0.01 ^a^	0.74 ± 0.04 ^a^
BEO	3.37 ± 0.25 ^b^	0.16 ± 0.02 ^a^	0.74 ± 0.03 ^a^

Data are the means ± SD. Different lowercase letters indicate significant differences, *p* < 0.05.

## Data Availability

Not applicable.

## Sample Availability

Samples of the compounds are not available from the authors.

## Abbreviations

ROS	reactive oxygen species
EOs	essential oils
CEOs	citrus essential oils
SEO	sweet orange essential oil
LEO	lemon essential oil
BEO	bergamot essential oil
GC-MS	gas chromatography-mass spectrometer
CON	control
FC	fatigue control
BLA	blood lactic acid
BUN	blood urea nitrogen
CK	creatine kinase
LG	liver glycogen
MG	muscle glycogen
LDH	lactate dehydrogenase
GSH-PX	glutathione peroxidase
MDA	malondialdehyde
SOD	superoxide dismutase
RT	retention time
ND	not detected
